# Enhancement of Sensitivity and Accuracy of Micro/Nano Water Droplets Detection Using Galvanic-Coupled Arrays

**DOI:** 10.3390/s19204500

**Published:** 2019-10-17

**Authors:** Rekha Goswami Shrestha, Tatsuya Ando, Yukihiro Sakamoto, Jin Kawakita

**Affiliations:** 1 Center for Functional Sensor & Actuator, Electrochemical Sensors Group, Research Center for Functional Materials, National Institute for Materials Science, 1-1 Namiki, Tsukuba, Ibaraki 305-0044, Japan; rekhashrestha3@hotmail.com; 2 Graduate School of Engineering, Chiba Institute of Technology, 2-17-1, Tsudanuma, Narashino, Chiba 275-0016, Japan; tatsuyaando77star@gmail.com (T.A.); yukihiro.sakamoto@it-chiba.ac.jp (Y.S.)

**Keywords:** galvanic current, electrodes, moisture, polymer, contact angle

## Abstract

A moisture sensor has been reported that detects invisibly small water droplets and distinguishes their particle size with high accuracy and high speed. This sensor uses narrow lines of dissimilar metals as electrodes, arranged with gaps of 0.5 to 10 μm. The working principle for this sensor is that it measures the galvanic current generated when a water droplet forms a bridge-like structure between the electrodes. In addition, the surface of the sensor was controlled by using hydrophilic polymer, GL, and hydrophobic polymer, PMMA. The study of the relationship between the contact angle, projected area of water droplets and current response from the sensor with a modified surface showed that in the case of GL, the contact angle was small (wettability increased) and the average value and distribution of the projected water droplet area and the sensor’s response increased. This enhanced the sensor’s sensitivity. On the other hand, in the case of PMMA, the contact angle was large (wettability decreased), the area of the water droplet and its distribution became small and the accuracy of discriminating the water droplet’s diameter by the sensor enhanced. Therefore, by rendering sensor’s surface hydrophilic and hydrophobic, the sensitivity and accuracy of the sensor could be enhanced.

## 1. Introduction

Small water droplets lead to various phenomenon like metal corrosion, glass fogging, molding, etc. Inevitable corrosion causes nuisance and economical losses of several orders in our daily life, such as in pipes used in transportation of underground water, gas and various products, electrical appliances, etc. Similarly, unavoidable glass fogging in vehicles, buildings, etc. causes invisibility leading to safety concerns. Likewise, molding can cause disease in many crops and make food products inedible, leading to an increase in food loss and countermeasure costs. The degree of all these phenomena are found to depend on the number and size of the water droplets. The smaller the water droplet is detected, in other words, in the earlier or more initial stage, the smaller the effect of these phenomena and energy for countermeasure such as dry-up is. Therefore, it is very important to detect the number and size of water droplets on the target in order to increase life time and functional evaluation of various products, for economical and safety concerns [1,2,3,4].

Small water droplets can be formed on the target by various condensation phenomenon, from water vapor contained in the atmosphere to water dew on the solid surface of the target. Using a hygrometer, dew condensation can be expected to occur on the solid surface thermodynamically as a result of relative humidity and temperature. Therefore, the measurement and control of humidity in the atmosphere is widely executed to avoid formation of small water droplets due to dew condensation in microelectronics, electronic devices, aerospace, meteorology, storage, and other fields [5,6,7,8,9,10]. However, most commercial hygrometers cannot detect actual dew condensation because their working principle is based on absorbing water molecules of vapor in the atmosphere with hygroscopic material, indicating that, according to this principle, a hygrometer cannot distinguish between vapor and liquid phases of water. Furthermore, in relation to this principle, it takes 10 s and more to reach stable values, and beyond 80% in relative humidity, the reliability of the values of most commercial hygrometers is decreased because changes in electrical value, such as resistance and capacitance of hygroscopic material by absorption of water molecules with relative humidity of around 80‒100%, become comparatively smaller than those around 20‒80%, leading to a decrease in accuracy of the relative humidity estimated from the electrical value. The diameter of water droplets can be determined using other commercial techniques, but most of them are mainly used for detection of relatively large droplets of more than 10 μm, corresponding to the lower detection limit of visual recognition, i.e., alternative to visible check. In addition, apparatus for these techniques are often very big in size, and multiple devices have to be fixed for their functional operation. Therefore, no commercial technologies exist to detect small water droplets yet. The commonly used condensation detectors like hygrometers, polymer condensation sensors and chilled mirror dew point hygrometers [11] need a long time to respond as many operational steps need to be passed through like mirror cooling, surface condensation, dew point calculation and other series of operations that consume extra time. They cannot distinguish between water droplets and water vapor that is condensed, and some cannot respond precisely at lower and higher relative humidity. The water droplet size plays a crucial role in measurement and control of condensation, and some cannot distinguish the smaller and bigger water droplets. They have many working components making them bigger in size and not suitable for simple measurements. Similarly, others require extra power supply for the operating components in order to detect output response [11,12]. 

In order to address all the above issues, the NIMS (National Institute for Materials Science) has developed a sensor to be able to detect a small water droplet of micro/sub-micro scale and to distinguish the particle diameter of minute water droplets with high sensitivity and quick response, and this sensor was termed a moisture sensor [13]. The principle of this sensor is that it measures the galvanic current generated across the electrodes, where a drop of water establishes a bridge-like structure. The electrodes are thin wires (arrays) of dissimilar metals arranged in gaps. By using a semiconductor fabrication process, the gap can be aligned to a relatively fine scale, such as micro/submicron size, leading to high detection sensitivity against small water droplets with such a diameter. The sensor can show an instantaneous signal upon introduction of water droplets on the sensor surface and signs off on complete evaporation, leading to quick response. By using these characteristics, this sensor is expected to detect dew condensation with high accuracy at an early or initial stage. Since the current response of this sensor should depend on the state (shape and size) of the water droplets present on the sensor surface, it is necessary to understand and establish the relation between the water droplet’s state and its corresponding current response. Moreover, the surface of the sensor can be coated and modified in order to improve the performance of the sensor (sensitivity and accuracy). 

The purpose of this research is to understand the relation between the water droplet’s state and its corresponding current response using simultaneous observation of the sensors surface and measurement of the sensor output. In addition, in order to show the performance of the sensor relatively, its sensing behavior of small water droplets was compared with a commercial hygrometer using different electrode gaps under a controlled humid condition. Furthermore, in order to improve the sensor characteristics, such as sensitivity and accuracy of the sensor against small water droplets, the wettability of the sensor’s surface was controlled by coating with hydrophilic and hydrophobic polymers.

## 2. Materials and Methods

### 2.1. Preparation of Sensor Chip, Surface Modification of Sensor Chip

**Figure 1:**
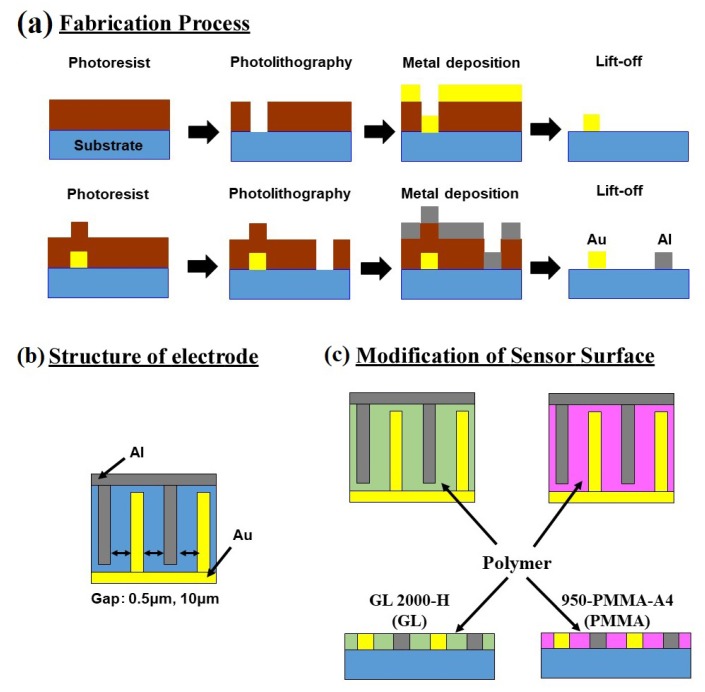
(**a**) Schematic flow of fabrication process of sensor with arrays of Al and Au electrodes on the sensor surface, (**b**) structure of electrodes and (**c**) modification of sensor surface by introduction of polymer PMMA and GL coatings onto the surface of the sensor (between the electrodes).

The sensor has an interdigit structure of electrodes made up of two different metals intercalating each other with a narrow gap in between each electrode. The sensor was made using the semiconductor microfabrication technique [13], as illustrated in Figure 1a. A silicon wafer of 4-inch diameter was used as the substrate. An insulating layer of silicon oxide was coated on the substrate surface using thermal oxidation. It was then treated by combining photolithography and metal deposition processes, to fabricate interdigit arrays made of gold (Au) and aluminum (Al) metals facing each other. The wafer processed was cut into chips of approximately 5 mm square. A schematic illustration of the arrays’ displacement is shown in Figure 1b. The gap between the adjacent arrays was set at 0.5 and 10 µm. The width and the thickness of the electrodes were 1 μm and 150 nm, respectively. The number of electrode pairs was 50. The surface modification between the electrodes was done using two types of polymers 950-PMMA-A4 (manufactured by MicroChem, abbreviated as PMMA) and GL2000-H (manufactured by Gluon Lab, abbreviated as GL). These polymers were introduced between the Al and Au electrodes by spin coating precursor of polymer, baking at 120 °C, and removing excess polymer on the electrode with plasma etching. The contact angle between the water droplets and the sensor surface was measured with a fully automatic contact angle meter (AST Products Inc./VCA Optima-XE).

### 2.2. Measurement of Sensor Output

The sensor chip was attached to a hand-made device (measure unit). This measurement unit is a custom-made device, which amplifies an analog signal from the chip and converts it to a digital signal. This signal was transmitted to a PC. Therefore, current was measured by the hand-made device putting a 20-bit, octal channel, current-input analog-to-digital (A/D) converter (Texas Instruments, DDC118) on board corresponding to currents from fA to µA range. The data was then collected as time-current data using the software on the PC. The output of this device was calibrated by using a semiconductor evaluation instrument (Agilent Technologies B1500A, Agilent Technologies, Tokyo, Japan). 

### 2.3. Introduction of Droplets on the Sensor Surface

**Figure 2:**
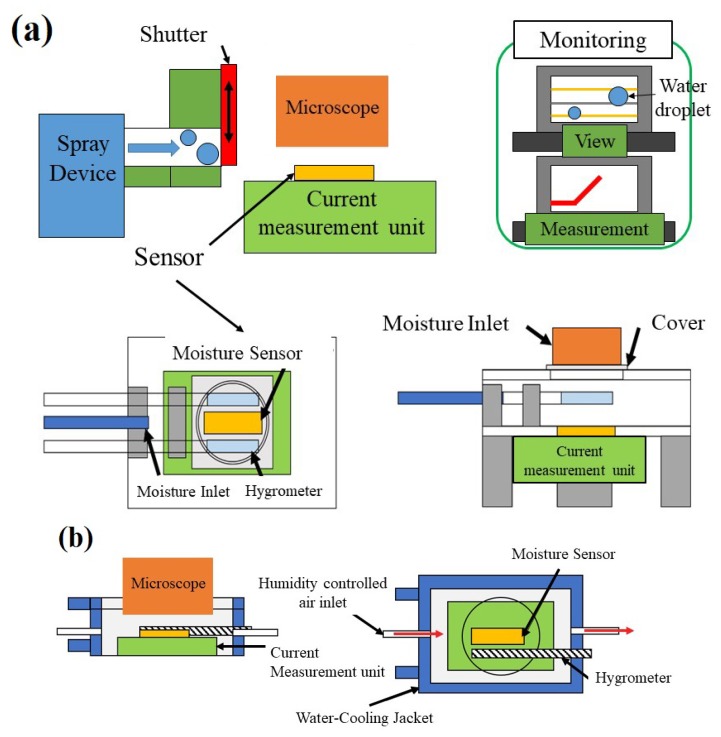
Schematic diagram showing an experimental setup for the introduction of a water droplet onto a moisture sensor (**a**) using a spray method and (**b**) by humidity-controlled air, while observing through a microscope and measuring the current.

The overall scheme of the experimental setup is shown in Figure 2. In the first case, the measurement device explained in the former section was placed in a chamber. The chamber was connected by a hose to a sprayer such as a nebulizer (OMRON/NE-U17, which sprays water droplets of particle size ~1–8 μm). A water droplet was introduced to the sensor surface using the sprayer. The humidity in the chamber was observed using a capacitive hygrometer (E+E Elektronic/EE23). The water used throughout this research was distilled water.

In the second case, the measurement device was placed in the other type of chamber. Its temperature could be controlled by the water circulation system installed within the chamber. Dew condensation on the sensor surface was induced by introducing humid air into the chamber. Humidity controlled air at 90% with a flow rate of 500 nccm (normal cubic centimeter per minute) was introduced into the water cooled chamber (at a steady state of 17 °C) for 180 s using a precise humidity control generator (Micro Equipment Inc., me-40DPRT-2FM-MFC). 

### 2.4. Observation of Surface of the Sensor

The sensor surface was observed using an optical microscope (KEYENCE Corporation/VHX-5000, Keyence Corporation, Osaka, Japan) from the top of the chamber using a digital camera (Figure 2) while the sensor output was collected.

## 3. Results and Discussion

### 3.1. Relation between Sensor Output and Droplet Status

**Figure 3:**
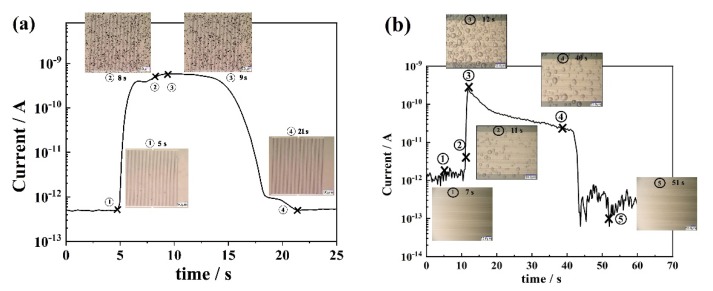
Current response on introduction of water droplets onto the sensor surface with time, and the corresponding microscopic images of the sensor surface at ① 5 s, ② 8 s, ③ 9 s and ④ 21 s of spraying in the case of figure (**a**) and at ① 7 s, ② 11 s, ③ 12 s, ④ 40 s and ⑤ 51 s of spraying in the case of figure (**b**).

Figure 3 shows the output current response and the corresponding microscopic images of the sensor surface, respectively, when water droplets are introduced onto the sensor surface by means of sprayer. The current response of the sensor quickly rose at 5 s when water droplets started to appear on the sensor surface (microscopic image ①). This is because water droplets on the surface of the sensor might form bridges between the two dissimilar electrodes, generating galvanic current. The moisture sensor was very fast in showing an output response in comparison to the hygrometer.

The sensor output, Figure 3a, attains almost maximum value at 8 s and keeps the same level of current up to 9 s. The corresponding microscopic image, ② showed that a larger number of water droplets covered a wider area on the sensor surface than before ①. Similar pattern was observed for 9s (microscopic images ③). Then, current drops rapidly to the background level at 21 s, while no water droplets were observed on the sensor surface (microscopic image ④), confirming a complete evaporation of the previously attached water droplets. Similar results were obtained for another set of moisture sensors under similar experimental conditions (Figure 3b). 

In conclusion, when there are no water droplets on the sensor’s surface, especially in between the electrodes, there is no output response from the sensor. The sensor instantaneously generates a current response due to the deposition/adhesion of minute water droplets in between the electrodes, and the current depends on the coverage status of the sensor surface, in other words, the bridging areas between the electrodes. This output response decreases to a background value due to disappearance of bridging water droplets by evaporation. This result also showed that the sensor had a quick response to the presence/disappearance of water droplets on the sensor surface.

### 3.2. Effects of Gap between Electrodes on Sensor Response

**Figure 4:**
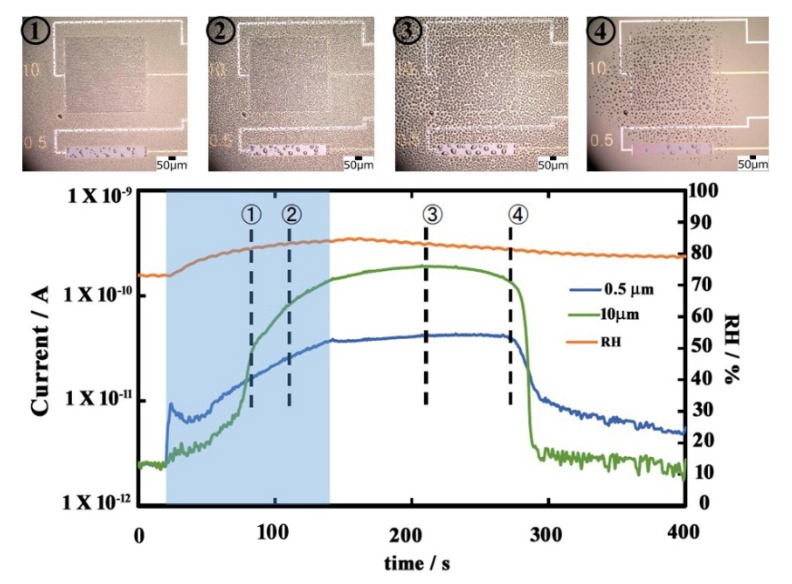
Current response of the sensors, and the corresponding microscopic images of the sensor surface with 0.5 μm and 10 μm gap between the electrodes on introduction of humidity-controlled air at different times.

From the above section, it has been proved that the moisture sensor shows a clear sensitivity and quick response compared to the hygrometer regarding the detection of small droplets. The sensing response from the sensor is due to the galvanic effects on introduction of a liquid bridge between the two dissimilar electrodes. Therefore, it is understandable that the gap between the two dissimilar electrodes plays an important role in the current response or the sensing performance of this sensor. The moisture sensor shows a clear sensitivity and quick response regarding detection of small droplets compared to the hygrometer. In the case of the moisture sensor, we can modify and adjust the gap between the electrodes to target actual droplet formation and control the sensing response. In order to evaluate the effects of spacing between the electrodes on its sensing response we studied sensors with two different spacings: 0.5 μm and 10 μm. Figure 4 shows the overall results of the sensing response and the corresponding microscopic images of the time-dependent variation on the sensor surface on introduction of water droplets onto the surface of these two types of sensors with time, at different relative humidity.

As shown in Figure 4, the current response from the sensor with 0.5 μm spacing (blue line) sharply increased immediately after 120 s from the introduction of humidity-controlled air. Water droplets were clearly observed on the sensor surface at 180 s (microscopic image ①) when the introduction of humid air was stopped. The sensor output regarding 0.5 μm spacing was gradually increased and showed a steady behavior after 240 s. In the range of current increase, the number of water droplets seems almost the same and the size becomes larger (microscopic images ① and ②), and in the range of steady current, the number and size of the water droplets seemed almost similar. On the other hand, the current value of the sensor with 10 μm spacing (black line) gradually increased only around 180 s of spraying, showing a peak value around 312 s, and then, a decreasing tendency was observed. The microscopic image regarding 10 μm spacing showed that the number of water droplets increased, followed by the increasing diameter of the water droplets (microscopic images ① to ③). A sharp decrease started at around 375 s for both the sensors. On introduction of humidity-controlled air, the sensor with 0.5 μm spaced electrodes showed a little bit faster current response in comparison to that with 10 μm spaced electrodes. When the humidity-controlled air touches the sensor surface, which is maintained at a lower temperature than the air, condensation occurs, forming water drops between the electrodes. When the electrode spacing is very narrow (0.5 μm), the bridge between the electrode is established a little earlier than that of the 10 μm spaced electrodes, where smaller droplets need to combine in order to form bigger droplets to bridge a wider space, for the output galvanic current response.

**Figure 5:**
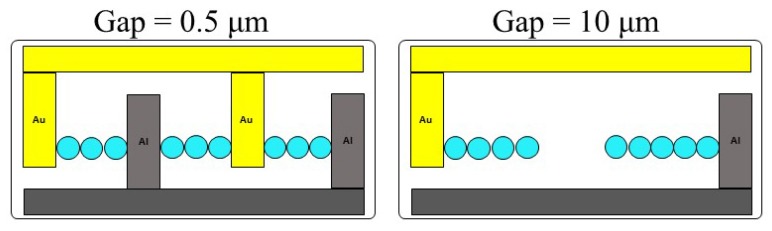
Schematic diagram showing the probable reason for the difference in the current response time for sensors with two different gaps between the electrodes.

The decreasing response is due to a decrease in the number of water droplets (microscopic images ④). The output response with condensation process on the electrodes was found to differ depending on the electrode spacing, as shown in the schematic diagram in Figure 5. 

The overall results suggest that the moisture sensor is very quick in responding to the water droplets (small size of 5 μm) attached to the sensor surface and the output response is very accurate compared to the ambiguous response from the commercial hygrometer. 

### 3.3. Influence of Surface Modification of Sensor on Its Response

The sensing response of the moisture sensor comes into play due to the generation of galvanic current when the water droplets form a bridge between the two dissimilar electrodes. Due to this bridging nature or capacity of liquid, ultimately the sensor response can be controlled by controlling the nature of the surface between the electrodes. One of the advantages of the moisture sensor is that we can modify and adjust the surface of this sensor to target actual droplet formation. There are several ways of modifying the surface of the sensor, such as ion beam sputtering [14], laser irradiation [15], plasma etching [16], etc. However, they have their own limitations, such as both the hydrophobic and hydrophilic surface cannot be obtained by a single operation. In this context, we modified the surface of the sensor by coating the surface with two types of polymers, hydrophilic, GL and hydrophobic, PMMA. Then, the output current response from the sensors without any coating and with GL and PMMA coatings was observed, by introducing water droplets onto the sensor surface with the sprayer.

**Figure 6:**
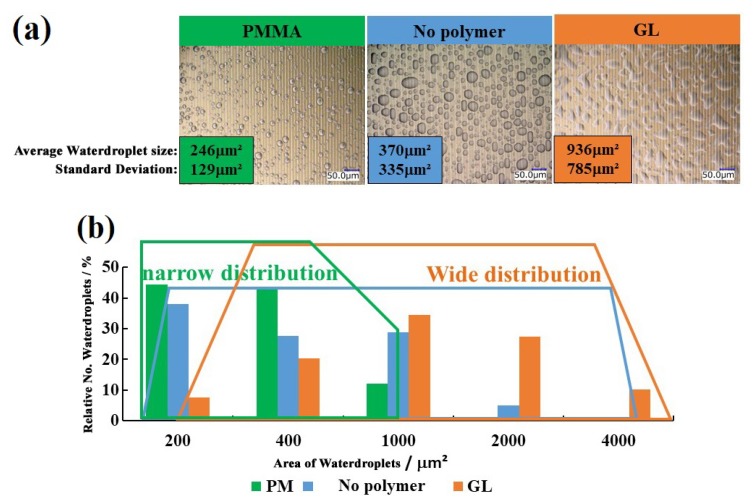
(**a**) The microscopic images of the sensor without polymer coating and coated with PMMA and GL; (**b**) the plot of relative number of water droplets against area of water droplets.

Figure 6a shows the microscopic images of the sensor surface with a 10 μm gap between the Al and Au electrodes without coating, and coated with GL and PMMA, when almost steady sensor current and droplet status on the observation were obtained. A clear difference can be seen among the in images of sensors with surface modification. From the microscopic images, the projected area of each droplet was estimated using computer software for image analysis (ImageJ). Figure 6b shows the distribution range of water droplets on the sensor surface. The sensor’s surface without polymer coating had wide distribution of thin and large water droplets ranging from 100 to 2000 μm^2^ (average particle size 370 μm^2^ and standard deviation 335 μm^2^). The PMMA coated sensor had round and small water droplets with narrow distribution from 50 to 1000 μm^2^ (average particle size 246 μm^2^ and standard deviation 129 μm^2^). In contrast to PMMA, most of the water droplets on the GL coated sensor were found to be comparatively large with wide distribution between 500 and 5000 μm^2^ (average particle size 936 μm^2^ and standard deviation 785 μm^2^).

**Figure 7:**
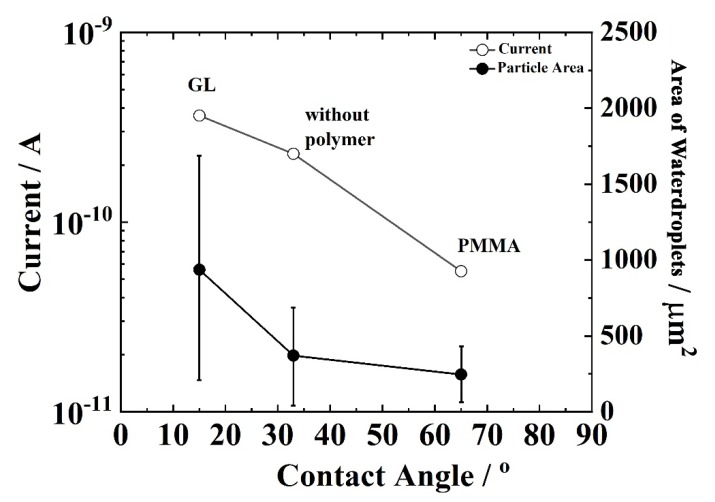
The output current and area of water droplets attached onto the sensor without polymer coating and coated with PMMA and GL as a function of contact angle between water droplets and the sensor surface area.

Figure 7 shows the current and area of water droplets as a function of contact angle with the sensor surface. It was found that the current response was in the following order: GL coated sensor > sensor without coating > PMMA coated sensor.

The nature of the surface determines the contact angle between the surface and the attaching liquid. This determines the shape and size of the drops on the surface, ultimately affecting the sensing response of the moisture sensor. The microscopic images in Figure 6a show that the average area of each water drop was the smallest in the case of PMMA, medium for the sensor without polymer, and largest for the GL.

When the surface was coated with the hydrophobic PMMA, the contact angle between the water droplets and the surface was found to be 65°, which suggests lower wettability. Smaller water droplets were observed after the spray. This hydrophobicity also helped the water droplets to maintain their shape and size. The accuracy of measurement was therefore better for hydrophobic PMMA than the other two. For the surface coated with the hydrophilic GL, the contact angle between the water droplets and the surface was found to be 15°, which suggests higher wettability. In the case of hydrophilic GL, bigger water droplets were observed after the spray and current enhancement was observed due to a larger increase in the number of contacts between the water droplets and the electrodes than for the other two. These results show that by controlling the nature of the sensor surface (hydrophilicity and hydrophobicity), we can successfully improve the detection sensitivity and accuracy of the sensor. In addition, this result suggests that the physicochemical status of the sensor surface can be adjusted to that of the target surface.

## 4. Discussion

A portable moisture sensor of very small size (~1 mm^2^) has been reported. It has two comb-like structures made of two dissimilar metals (Al and Au, in this study) that are intercalated with each other. The gap between the electrodes can be fixed according to our requirements (0.5 and 10 μm, in this study). This gap can in fact give us information about the shape and size of water droplets adhered in between the electrodes. This moisture sensor works on a very simple principle: the generation of galvanic current on formation of a water droplet bridge between the two different electrodes. This study has shown that we can observe the sensor’s surface and also measure the current output from the sensor simultaneously. This helps in predicting and explaining all the probable cause and phenomena occurring during dew condensation. The results (current vs. time) clearly shows an immediate current response due to the adhesion-evaporation process of water droplets (>20 ms). A very small water droplet (0.5 μm, in this study) can be detected quickly and accurately. Additionally, the gap between the electrodes can be varied according to our requirements, thus making it precise in predicting the shape and size and also the number of water droplets. The output basically corresponds to the adhesion/evaporation of water droplets. Additionally, modifying the surface of the sensor with hydrophilic and hydrophobic polymers could enhance the detection sensitivity and distinction accuracy for smaller water droplets.

This research has successfully reported an optimized experimental set up for a simultaneous current measurement and surface observation with a moisture sensor. Therefore, a quick and accurate response of sensor output as a result of the attachment of water droplets can be realized. The response time of the moisture sensor is fast enough to detect initial condensation and therefore can be applied in the automobile industry, food industry and several other infrastructures. This sensor and the polymer modified sensor show selectivity to the shape and size of the water droplets, meaning that this sensor can be used to distinguish water droplets (moisture) and therefore can be used in the fields of beauty and healthcare, with greater success than the commercial hygrometer, which cannot detect droplet and distinguish its size.

## 5. Conclusions

A small moisture sensor (~1 mm^2^) with two dissimilar metals (Al and Au) arranged in an interdigit structure reported in this study clearly shows an instantaneous current response due to the adhesion-evaporation process of water droplets. The main principle on which the sensor works is that when water droplets form a bridge between the two different electrodes, galvanic current is generated, giving current response.

The main conclusions of this study can be summarised as follows:

1. Simultaneous observation of the sensor surface and measurement of sensor output showed that the moisture sensor can respond to the presence/disappearance of small water droplets around 0.5 μm quickly and accurately.

2. The use of different gap sizes between the electrodes showed high sensitivity against the attached water droplets and reflected changes in the number and shape of the droplets, compared to the commercial hygrometer.

3. Modification of the surface status of the sensor, in terms of wettability, can enhance the detection sensitivity and distinction accuracy of the sensor for small water droplets. In addition, sensor output is expected to get close to the target surface by controlling the surface status.
